# Development and validation of a nomogram to predict severe influenza

**DOI:** 10.1002/iid3.70026

**Published:** 2024-09-28

**Authors:** Mingzhen Zhao, Bo Zhang, Mingjun Yan, Zhiwei Zhao

**Affiliations:** ^1^ Pulmonary and Critical Care Medicine Affiliated Hospital of Chengde Medical University Chengde Hebei China

**Keywords:** duration of illness, haptoglobin, myeloperoxidase, nomogram, severe influenza

## Abstract

**Background:**

Influenza is an acute respiratory disease posing significant harm to human health. Early prediction and intervention in patients at risk of developing severe influenza can significantly decrease mortality.

**Method:**

A comprehensive analysis of 146 patients with influenza was conducted using the Gene Expression Omnibus (GEO) database. We assessed the relationship between severe influenza and patients' clinical information and molecular characteristics. First, the variables of differentially expressed genes were selected using R software. Least absolute shrinkage and selection operator (LASSO) and multivariate logistic regression analysis were performed to investigate the association between clinical information and molecular characteristics and severe influenza. A nomogram was developed to predict the presence of severe influenza. At the same time, the concordance index (*C*‐index) is adopted area under the receiver operating characteristic (ROC), area under the curve (AUC), decision curve analysis (DCA), and calibration curve to evaluate the predictive ability of the model and its clinical application.

**Results:**

Severe influenza was identified in 47 of 146 patients (32.20%) and was significantly related to age and duration of illness. Multivariate logistic regression demonstrated significant correlations between severe influenza and myloperoxidase (MPO) level, haptoglobin (HP) level, and duration of illness. A nomogram was formulated based on MPO level, HP level, and duration of illness. This model produced a *C*‐index of 0.904 and AUC of 0.904.

**Conclusions:**

A nomogram based on the expression levels of MPO, HP, and duration of illness is an efficient model for the early identification of patients with severe influenza. These results will be useful in guiding prevention and treatment for severe influenza disease.

## Graphical abstract

We developed and validated a nomogram to predict severe influenza. By the nomogram, early prediction, and intervention for patients who may develop severe influenza will greatly reduce their mortality and sequelae.

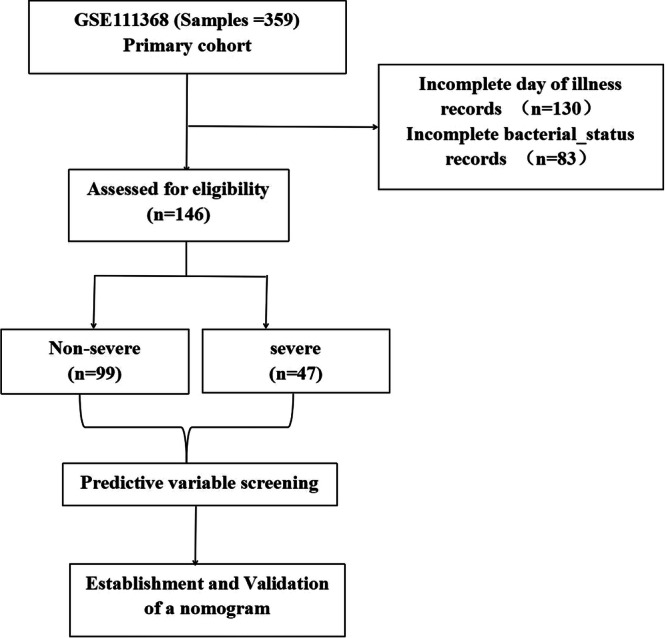

## 1 BACKGROUND

Influenza viruses are among the most widespread and contribute to pandemic viruses worldwide, demonstrating a catastrophic impact on human health.^1^ The World Health Organization reported that the annual incidence of influenza in adults is 10% globally, and the annual incidence of influenza in children is 20%–30%.^2^ It is worth noting that influenza outbreaks can cause 3,000,000–5,000,000 severe cases and 290,000–650,000 deaths each year.^3^ Some severe cases are prone to viral pneumonia, acute kidney injury, myocardial injury, multiple organ failure, and disseminated intravascular coagulation, which contribute to high mortality.^4^, ^5^, ^6^ These factors have caused a global burden on health, society, and the economy.^7^, ^8^, ^9^ Patients with mild influenza often miss opportunities for intervention and treatment, because physicians cannot predict whether mild influenza will develop into a severe influenza at an early stage. Therefore, early detection of patients at risk of transitioning to severe symptoms, timely strengthening surveillance, and effective treatment can help improve patient prognosis.

Although several studies have assessed the risk factors for severe influenza, none have been universally accepted for individualized prediction based on scientific evidence.^10^, ^11^, ^12^, ^13^ As a visual predictive tool, nomograms calculate individual risk of outcomes and have recently been widely applied to evaluate disease prevention.^14^, ^15^, ^16^, ^17^ To help clinicians identify severe influenza early and reduce morbidity and mortality of severe influenza, we developed and validated a nomogram of severe influenza from an influenza cohort.

## 2 METHODS

### 2.1 Data collection and study population

Ribonucleic acid (RNA) sequence data (GSE111368) was obtained from the public Gene Expression Omnibus (GEO) Dataset (https://www.ncbi.nlm.nih.gov/geo/). A total of 359 patients were downloaded from the database, of which 130 lacked disease duration of illness and 83 lacked bacterial infection data. These data are excluded. Finally, 146 eligible patients were selected for analysis. The severity of influenza is defined according to whether invasive mechanical ventilation is needed. Patient characteristics influenza type, duration of illness, gender, age, ethnicity, bacterial status) were analyzed. The expression data sets of blood samples are achieved by Microarray Gene Expression Profiling after RNA extraction via the R software.

### 2.2 Identification of differentially expressed genes (DEGs)

We used the expression data sets of blood samples from GSE111368 to identify DEGs in patients with severe and nonsevere influenza via the “limma” package in the R software,^18^ with a false discovery rate (FDR) < 0.05 and a log2|fold change|> 2 as cutoff values. Eighteen DEGs were selected in the volcano plot in the R software.

### 2.3 Variables of influenza patients with severe infection

We combined patient clinical data with DEGs. First, univariate least absolute shrinkage and selection operator (LASSO) regression analysis was used to analyze and screen for the variables leading to severe influenza. Ten‐fold cross‐validation was adopted and R software was used to centralize and normalize the variables. Then, multiple logistic regression analyses were performed to determine the final predictors of the results of the univariate regression analysis.

### 2.4 Development and validation of a nomogram of severe influenza

Based on multivariate logistic regression analysis, we constructed a nomogram of severe influenza using the “Regression Modeling Strategies” (rms) package in R software. The nomogram included three variables: duration of illness, haptoglobin (HP) level, and myloperoxidase (MPO) level. Then, we evaluate the predictive ability of the established model using the concordance index (*C*‐index) and area under the curve (AUC) of receiver operating characteristic (ROC). The *C*‐index is used to assess the accuracy and reliability of the nomogram established, and the area under the ROC curve is used to assess the ability of the model to predict severe influenza patients. The clinical application ability of the nomogram was analyzed by decision curve analysis (DCA) and clinical impact curves (CICs). The analysis software is R4.2.1.

## 3 RESULTS

### 3.1 Patient characteristics

**Figure 1:**
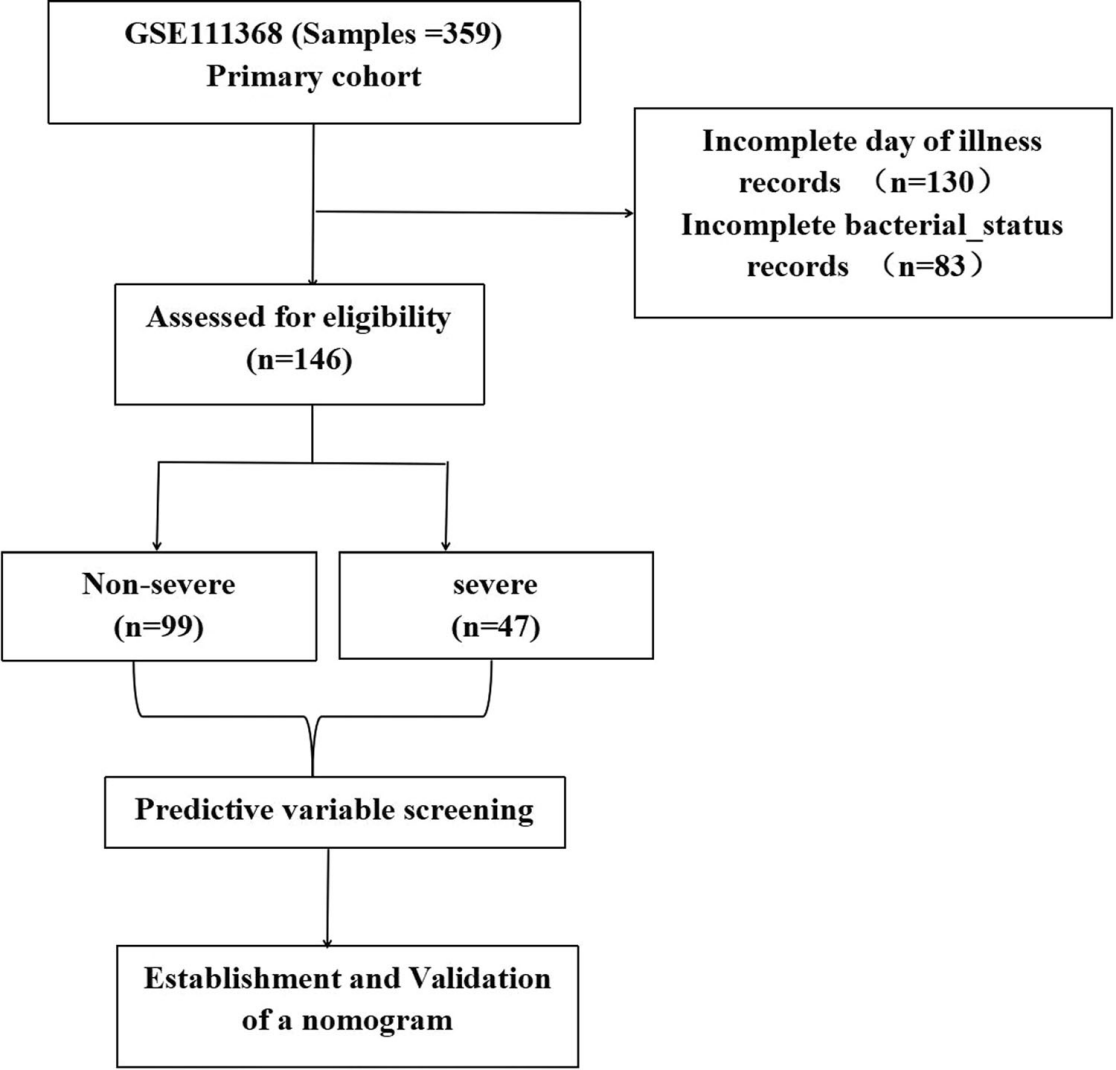
Flow diagram of data collection and analysis.

In total, 146 patients were included in this study (Figure 1). Among these patients, 47 had severe disease and 99 had nonsevere disease. The participant characteristics included influenza type, sex, age, duration of illness, ethnicity, and bacterial status (Table 1). The age was 44 (range: 31–52) and 40 (range: 28–44) years and the mean duration of illness was 8 (range: 5–16) and 12 (range: 8–17) days in the non‐severe and severe group, respectively. It was statistically significant when *p* < .05, and the variables were included in the analysis.

**Table 1:** Baseline characteristics of study patients with influenza.

	Data set (*n* = 146)		
Study variables	Non‐severe = 0 (*n* = 99)	Severe = 1 (*n* = 47)	*p* Value*
Flu type			0.295
H1N1	80 (81)	44 (94)	
A (unknown)	2 (2)	NA	
B (unknown)	15 (15)	3 (6)	
H3N2	2 (2)	NA	
Gender, No. (%)			0.732
Male	53 (54)	23 (49)	
Female	46 (46)	24 (51)	
Age (years), median (IQR)	44 (31, 52)	40 (28, 44)	0.012
Duration of illness, Median (Q1, Q3)	8 (5, 16)	12 (8, 17)	0.009
Ethnicity no. (%)			0.242
White	73 (74)	32 (68)	
Black	12 (12)	7 (15)	
Asian	9 (9)	8 (17)	
Other	5 (5)	NA	
Bacterial status			0.091
Yes	49 (49)	31 (66)	
No	50 (51)	16 (34)	

*Note*: Data presented as median (IQR) or numbers, with percentages in parentheses*, Mann–Whitney *U* test was used for the continuous variables and Fisher's exact test was used for categorical variables specified in the exact argument.

Abbreviations: IQR, interquartile range; NA, not applicable.

### 3.2 Identification of DEGs

**Figure 2:**
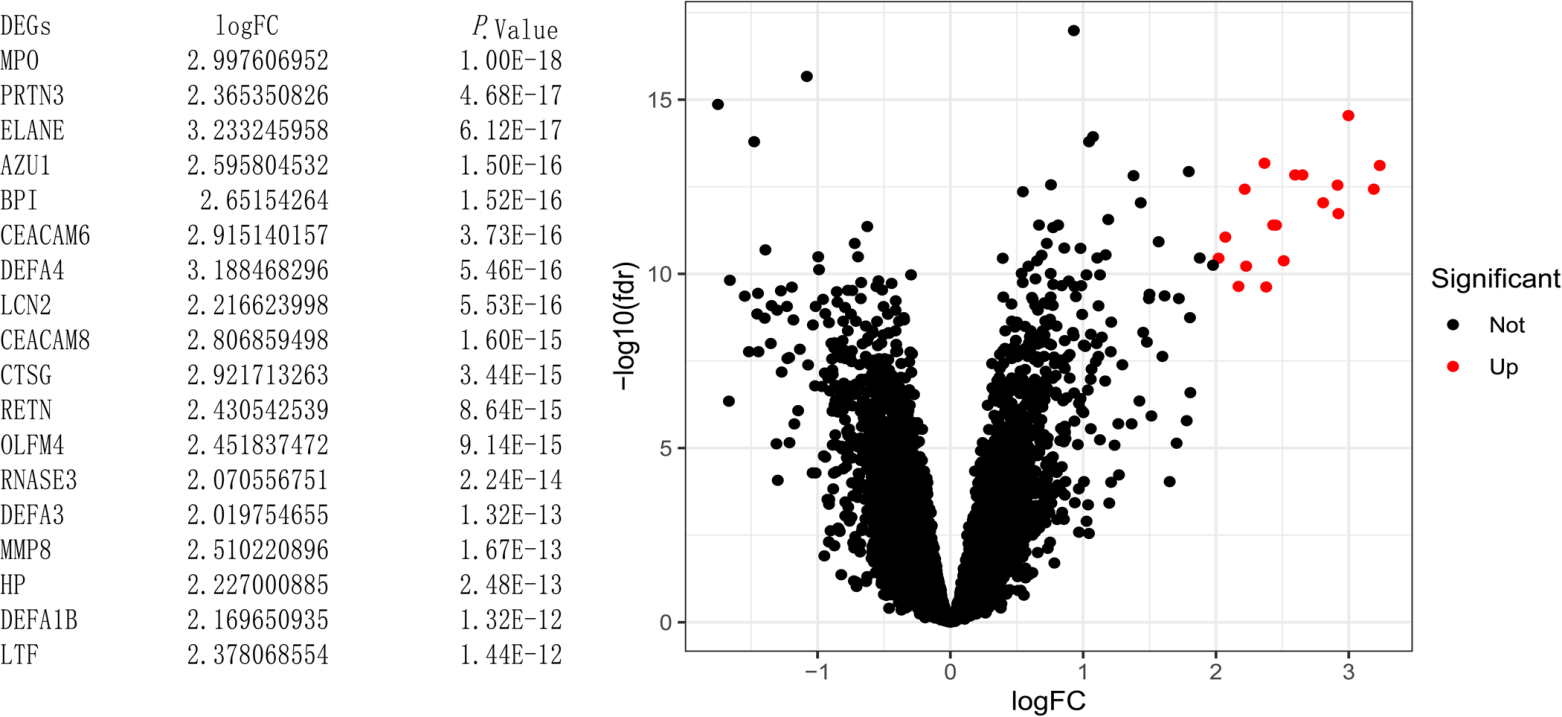
Volcano map of DEGs. Red spots represent upregulated genes.

Expression data sets of the blood samples were obtained by Microarray Gene Expression Profiling after RNA extraction. The expression data set was analyzed to identify DEGs between non‐severe and severe influenza using R software, with FDR < 0.05, and a log2|fold change|> 2 as cutoff values. A total of 18 DEGs (MPO, PRTN3, ELANE, AZU1, BPI, CEACAM6, DEFA4, LCN2, CEACAM8, CTSG, RETN, OLFM4, RNASE3, DEFA3, MMP8, HP, DEFA1B, and LTF) were screened in the volcano plot in the “ggplot2” package in R software (Figure 2).

### 3.3 Selected variables for the nomogram

**Figure 3:**
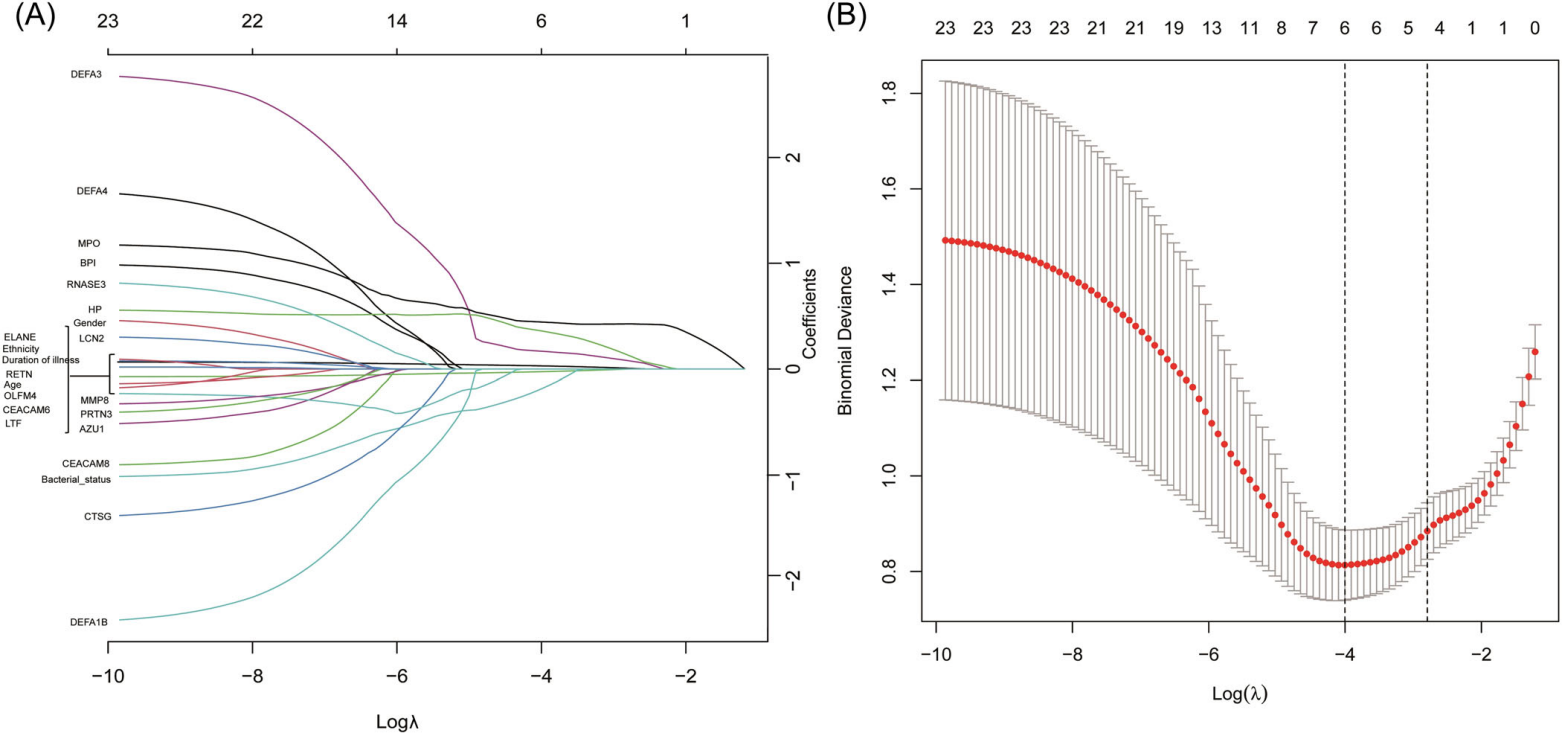
Variables selection using least absolute shrinkage and selection operator (LASSO) regression analysis. (A) Log*λ* and partial likelihood deviations are shown and the dotted line shown at the smallest log*λ* represents the best predictor. (B) LASSO coefficients for the six Variables. Non‐zero coefficients were determined based on the best log*λ*.

To select variables that predict severe influenza, patient characteristics (influenza type, age, duration of illness, sex, ethnicity, and bacterial status), and DEGs (MPO, PRTN3, ELANE, AZU1, BPI, CEACAM6, DEFA4, LCN2, CEACAM8, CTSG, RETN, OLFM4, RNASE3, DEFA3, MMP8, HP, DEFA1B, and LTF) were included in the LASSO regression analysis. LASSO analysis showed that severe influenza was significantly correlated with duration of illness, age, bacterial status, DEFA3, HP, and MPO (Figures 3A,B). Based on the results of the LASSO regression analysis, we conducted a multiple logistic regression analysis. Three variables were obtained: duration of illness, MPO, and HP (Table 2).

**Table 2:** Multivariate logistic regression analysis of predictors selected by LASSO regression procedure in the data set.

Independent variables	Multivariable logistic regression analysis	
	OR (95% CI)	*p*‐Value
Duration of illness	1.047 (1.019–1.079)	.001**
Age	0.956 (0.910–1.001)	.063
Bacterial status	0.577 (0.182–1.761)	.337
DEFA3	1.287 (0.699–2.664)	.452
HP	1.668 (1.030–2.751)	.038*
MPO	1.625 (1.037–2.645)	.040*

*Note*: Significant codes **0.01, *0.05.

Abbreviations: CI, confidence interval; DEFA3, defensin, alpha 3; HP, haptoglobin; LASSO, least absolute shrinkage and selection operator; MPO, myeloperoxidase; OR, odd ratio.

### 3.4 Developed predictive nomogram of severe influenza

**Figure 4:**
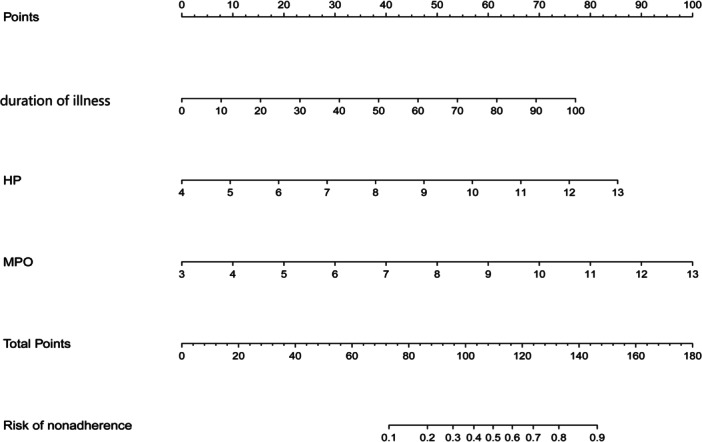
The use of a nomogram to determine the probability of severity of influenza. A nomogram for severity was designed and assimilated with the variables The variable's points were found on the uppermost point scale that matched with each patient variable and was added. The total points extrapolated to the bottom scale show the percent probability of severity.

Based on the logistic regression analysis, a nomogram was developed for the three variables predicting severe influenza (Figure 4). On the left side of Figure 4, the items shown from top to bottom are: “Points,” the name of the variables, “Total points” and “Risk.” The lines corresponding to each item are marked with a scale. The contribution of clinical outcomes for each variable is represented by the length of the line segment. The “Points” in the figure represent the score for each variable corresponding to the condition. “Total points” represents the total score of all variables assigned to a patient. “Risk” shows the predicted probability of severe influenza. As a result, we developed this simple prediction tool for patients with severe influenza. The total score was calculated based on disease duration, MPO, and HP. The scores for each variable are assigned on the point scale axis, and by adding the scores for each variable, we can easily calculate an overall score that shows the risk for each patient. Thus achieving an estimate of the presence of severe influenza.

### 3.5 Validated predictive nomogram for the probability of severe influenza

**Figure 5:**
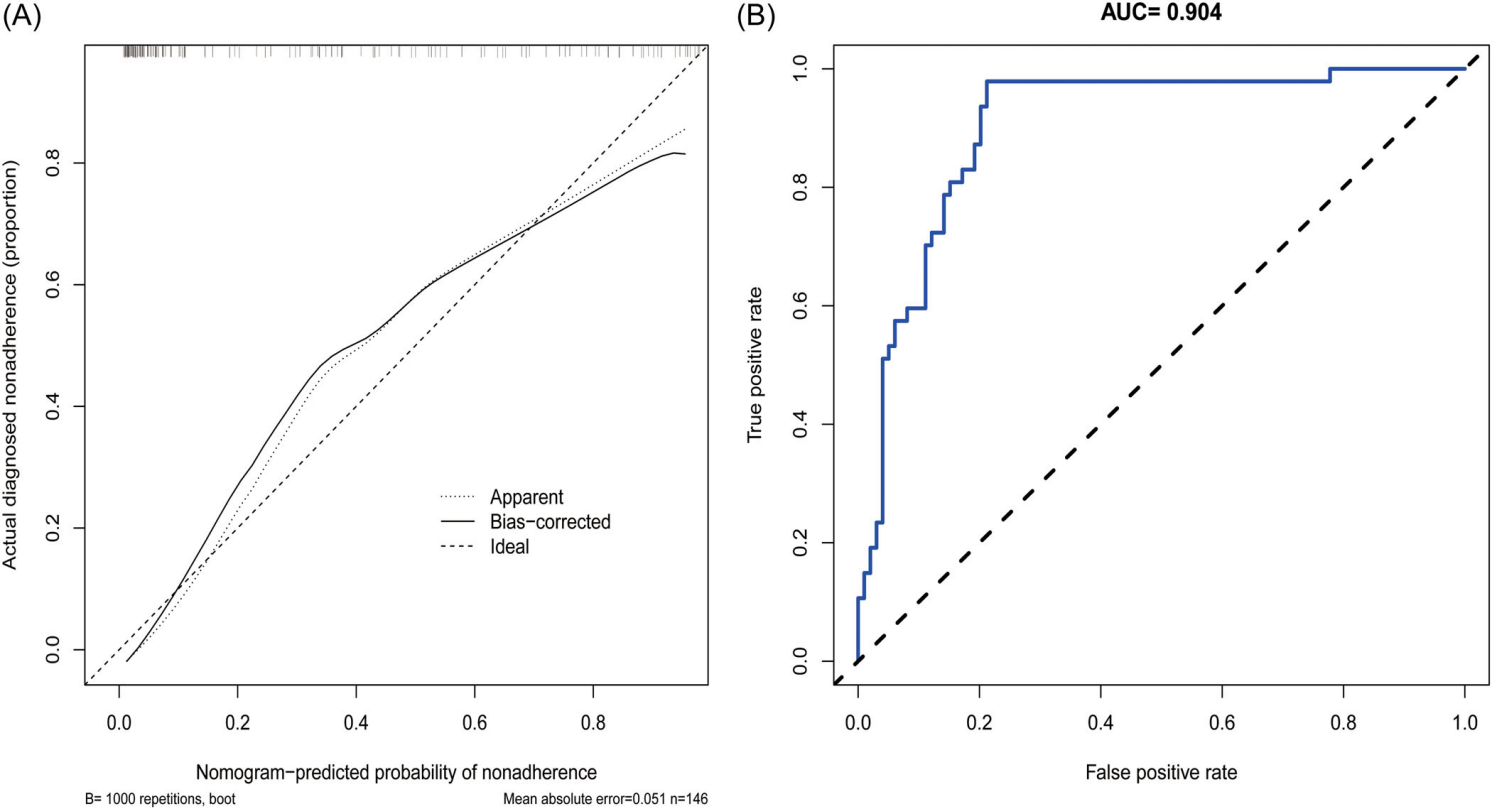
Evaluation of the nomogram. (A) Calibration plots of the predictive model Calibration plot in the data set. The *x*‐axis is the predicted probability of the severity of influenza. The *y*‐axis is the observed severity of influenza. The diagonal dotted line represents a perfect prediction by an ideal model. The solid line represents the performance of the nomogram. It represents a better prediction that a solid line is close to a diagonal dotted line. The figure shows that the prediction model has a good predictive ability. (B) Receiver operating characteristic (ROC) curve with area under the curve (AUC = 0.904) based on the predictive nomogram. The nomogram exhibited excellent power of discrimination with an AUC of 0.904 in the data set.

The calibration curves were used to evaluate the accuracy and reliability of the constructed nomogram. The bootstrap self‐sampling technique was used to repeat 1000 times. As shown in Figure 5A, the predicted probability of severe influenza showed a high consistency with the actual probability. Where the *X*‐axis shows the predicted probability and the *Y*‐axis showed the actual probability of severe influenza. The dotted lines for the diagonals of the figure show the perfect model of prediction under ideal conditions. The solid line shows the performance of the nomogram we built. The closer the diagonal between the solid line and the dotted line, the better the prediction performance. Notably, the *C*‐index was 0.904 [95% confidence interval = 0.802–1.006] and the AUC was 0.904 for predicting severe influenza in the database (Figure 5B). Taken together, these findings suggest that the nomogram for severe influenza had accurate discriminative and calibration abilities.

### 3.6 Clinical value of the nomogram

**Figure 6:**
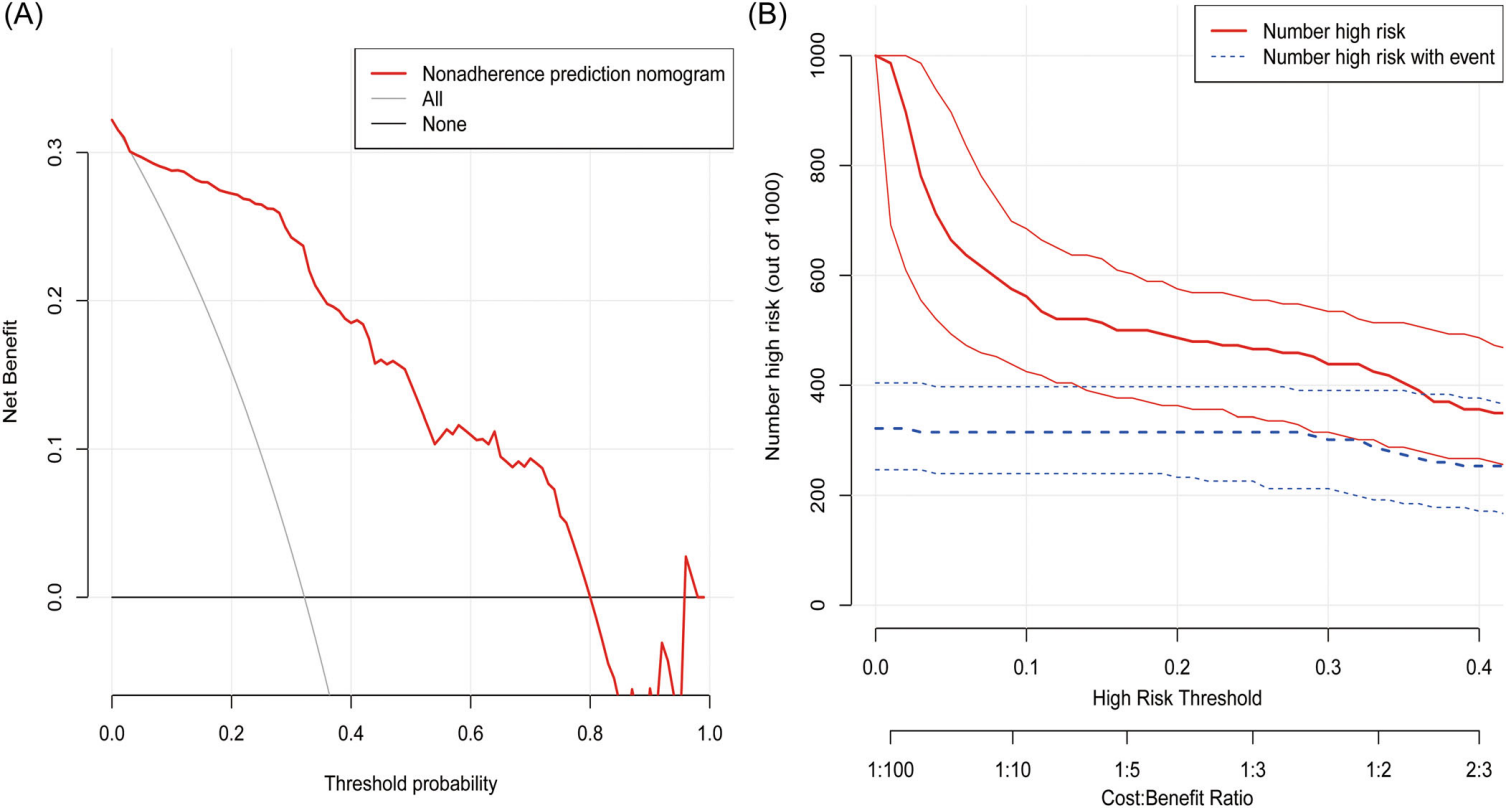
The clinical use of the nomogram. (A) Decision curve analysis for the nomogram. The red curve is the benefit predicted by the model, the gray curve is the benefit rate if all patients receive the intervention, and the horizontal line is the benefit rate if all patients receive no intervention. The intersection of the red curve with all is taken as the starting point, and the intersection of the red curve with none is taken as the end. Patients in this range can benefit. (B) Clinical impact curve (CIC) for predicting the severity of influenza. The red curve (number of high‐risk individuals) indicates the number of people who are classified as positive (high risk) by the model at each threshold probability; the blue curve (number of high‐risk individuals without come) is the number of true positives at each threshold probability. CIC visually indicated that the nomogram conferred high clinical net benefit and confirmed the clinical value of the model.

The DCA and CICs were evaluated for clinical use of nomogram. As shown in Figure 6A, the red curve showed the benefit of using the nomogram, the gray curve showed the benefit rate for all patients with intervention, and the horizontal line shows the benefit rate for all patients without intervention. In our study, DCA results showed that nomogram intervention was more beneficial than intervention or nonintervention for all patients, with a threshold probability of 0.01 to 0.8. The CICs results were shown in Figure 6B, with probability thresholds on the *X*‐axis and a number of patients on the *Y*‐axis. The blue lines represent the number of patients identified by the model as high risk at different probability thresholds. The red lines represent the number of patients identified by the model as high risk when experiencing clinical outcomes at different probability thresholds. The results of both DCA and CICs indicate that the nomogram is promising and of great clinical value in predicting severe influenza.

## 4 DISCUSSION

Patients with severe influenza still have high mortality rates.^19^, ^20^ Previous studies have demonstrated that the best prevention strategy is to identify individuals at risk of developing the disease at an early stage.^21^, ^22^, ^23^, ^24^ As visual prediction tools, nomograms can be used to estimate individual outcome risks early. Nomograms have been used to make the diagnosis and prognosis of various diseases. However, nomograms are rarely used as predictive models for severe influenza. The accurate prediction and estimation of patients with severe influenza are essential for patient counseling and treatment decision‐making. Therefore, in this study, we developed and validated a nomogram that accurately predicted the probability of severe influenza in the early stages. Therefore, this can enhance early decision‐making by clinicians and patients to gain more net benefits and reduce mortality rates.

Among the three predictors, the duration of illness was the one with the largest risk. Indeed, Pamela et al. confirmed that the duration of illness correlates with the degree of pathological damage in ferrets infected with influenza virus.^25^ At 4–7 days postinfection, animals with severe illness showed a significant decrease in body weight compared to animals in the nonsevere group. Early treatment of patients with influenza can reduce the risk of bacterial and fungal infection^26^, ^27^, ^28^, ^29^ and various complications.^30^, ^31^, ^32^, ^33^, ^34^ The benefit of influenza treatment is optimal when antiviral therapy is started within 24–48 h of symptom onset because drugs not only decrease virus replication but also decrease the risk of serious complications by intervening early.^35^ Oseltamivir, the most commonly prescribed and effectively utilized neuraminidase inhibitor for treating influenza infections, is an orally administered antiviral drug approved under the original trade name Tamiflu® (Roche). However, treatment with oseltamivir is limited by the duration of illness^36^, ^37^, ^38^; oseltamivir therapy within 2 days of symptoms onset is associated with a reduction in influenza‐related deaths. Therefore, illness duration is a significant risk factor for prognosis.^35^

MPO, also known as peroxidase, is a heme‐containing coenzyme and a member of the heme peroxidase superfamily.^39^ Notably, MPO can produce highly harmful reactive oxygen species (ROS) and hypochlorous acid (HOCl) using hydrogen peroxide (H_2_O_2_) and chloride ions as substrate.^40^ In addition, MPO is closely associated with coronary artery disease, rheumatoid arthritis, asthma, cerebral ischemia–reperfusion injury, interstitial lung disease, and cancer.^41^, ^42^, ^43^, ^44^, ^45^, ^46^ Several studies have shown that MPO can aggravate disease in a mouse model of influenza virus infection through the role of MPO‐ROS‐generating enzymes.^47^, ^48^, ^49^ DeLa et al. showed that influenza A virus‐induced ROS, pro‐inflammatory mediators, and cell death are closely related to increased MPO levels.^50^ Additionally, Phung et al. suggested that PR‐8 (A/H1N1) may cause a cytokine storm in the presence of an H(2)O(2)‐MPO system.^51^ Similarly, serum MPO levels are significantly elevated in patients with ARDS and H5N1 infection.^52^ Furthermore, several studies have proposed that MPO is an independent risk factor for the early prediction of severe influenza.^53^

HP, which binds to hemoglobin (Hb) in plasma,^54^ is located on chromosome 16 in the form of three alleles.^55^, ^56^, ^57^ The liver and lungs are the primary sites of HP synthesis.^58^, ^59^ HP is a complex protein that plays multiple roles in various biological processes. Under physiological conditions, Hb in the serum is cleared from the circulatory system by binding to macrophage‐specific receptors.^60^ However, in pathological conditions, such as certain hemolytic diseases, HP tightly combines with Hb during hemolysis.^61^, ^62^

The formation of Hb–HP complexes reduces the oxidative properties of Hb. Subsequently, the exhaustion of serum HP causes oversaturation of free Hb in the plasma. In addition, free Hb can cause pathological damage in vivo by stimulating lipid peroxidation and the immune response. Studies have reported that the HP concentration may increase substantially during acute infectious diseases.^63^, ^64^ Thus, HP is considered a potential biomarker for the diagnosis of many diseases.^65^, ^66^, ^67^, ^68^ In addition, several studies have shown that HP can lead to significant lung injury and that the HP plasma concentration is an optimal biomarker during acute viral infection.^69^, ^70^

In summary, we developed a nomogram to predict severe influenza using an influenza data set. The nomogram model, based on the expression levels of MPO and HP and the duration of illness, was verified as a reliable prediction model for severe influenza.

Although the established prognostic nomogram has a good ability to predict severe disease in patients with influenza, our study has certain limitations. First, influenza‐related information was derived from a public database; therefore, an external cohort of clinical samples was collected for verification. Second, because publicly available data are limited, the medical histories of patients were not comprehensively analyzed. Third, the sample size was relatively small, and we did not divide the samples into training and validation cohorts. In the future, more detailed and complete prospective cohort studies with larger sample sizes should be designed to further improve the accuracy of our predictive nomogram.

## 5 CONCLUSION

The nomogram for severe influenza, based on the expression levels of MPO and HP and duration of illness, was demonstrated to have good discrimination, calibration, and clinical value.

## AUTHOR CONTRIBUTIONS

Zhiwei Zhao contributed to the design and provided guidance for this study. Mingzhen Zhao screened the data in the GEO database and completed the bioinformatics analysis. Mingjun Yan performed the statistical analysis and visualization. The initial draft of the manuscript was written by Bo Zhang and Mingzhen Zhao, who revised the manuscript.

## CONFLICT OF INTEREST STATEMENT

The authors declare no conflict of interest.

## Data Availability

The original microarray data and related information are available at the Gene Expression Omnibus (Accession number: GSE111368; https://www.ncbi.nlm.nih.gov/geo/).
